# Development and Optimization of Nano-Hydroxyapatite Encapsulating Tocotrienol-Rich Fraction Formulation Using Response Surface Methodology

**DOI:** 10.3390/pharmaceutics17010010

**Published:** 2024-12-24

**Authors:** Anis Syauqina Mohd Zaffarin, Shiow-Fern Ng, Min Hwei Ng, Haniza Hassan, Ekram Alias

**Affiliations:** 1 Department of Biochemistry, Faculty of Medicine, Universiti Kebangsaan Malaysia, Jalan Yaacob Latif, Bandar Tun Razak, Kuala Lumpur 56000, Malaysia; anissyauqina96@gmail.com; 2 Faculty of Pharmacy, Universiti Kebangsaan Malaysia, Jalan Raja Muda Abdul Aziz, Kuala Lumpur 50300, Malaysia; nsfern@ukm.edu.my; 3 Department of Tissue Engineering and Regenerative Medicine, Faculty of Medicine, Universiti Kebangsaan Malaysia, Jalan Yaacob Latif, Bandar Tun Razak, Kuala Lumpur 56000, Malaysia; angela@ukm.edu.my; 4 Department of Human Anatomy, Faculty of Medicine and Health Sciences, Universiti Putra Malaysia, Serdang 43400, Malaysia

**Keywords:** response surface methodology, central composite design, nano-hydroxyapatite, tocotrienol-rich fraction, nanoformulation, nanocarrier

## Abstract

**Background/Objective**: The tocotrienol-rich fraction (TRF) is a lipid-soluble vitamin that has good antioxidant and anti-inflammatory properties. The TRF is widely studied as a potential treatment for various diseases, including bone diseases. However, its application is limited due to its poor oral bioavailability profile, warranting an innovative approach to overcome its pharmacokinetic limitations. Recently, the nano-hydroxyapatite (nHA) has been investigated as a drug delivery vehicle for various drugs and active compounds owing to its excellent biocompatibility, biodegradability, and osteogenic properties. The nHA is also a well-known biomaterial which has chemical and structural similarities to bone minerals. Hence, we aim to explore the use of the nHA as a potential nanocarrier for the TRF. **Methods**: In this study, we develop and optimize the formulation of an nHA-encapsulating TRF (nHA/TRF) by employing the response surface methodology (RSM). **Results**: RSM outcomes reveal that the mass of the nHA, the concentration of the TRF, and the incubation time have a significant effect on the particle size, zeta potential, and encapsulation efficiency of the nHA/TRF. The outcomes for the optimized formulation are not significantly different from the predicted RSM outcomes. The optimized nHA/TRF formulation is freeze-dried and results in an average particle size of ~270 nm, a negative zeta potential value of ~−20 mV, a polydispersity index of <0.4, and an encapsulation efficiency of ~18.1%. Transmission electron microscopy (TEM) shows that the freeze-dried nHA/TRF has a spherical structure. **Conclusions**: Taken together, the above findings indicate that the nHA may be established as a nanocarrier for efficient delivery of the TRF, as demonstrated by the promising physical properties.

## 1. Introduction

Vitamin E is a large family of lipid-soluble vitamins, composed of tocopherols and tocotrienols. Each group is further subdivided into four different isomers: alpha (α), beta (β), gamma (γ), and delta (δ). Tocopherols and tocotrienols are well-known for their excellent antioxidant and anti-inflammatory properties, and have thus attracted great interest among researchers interested in investigating their potential application to nutraceuticals [1]. Interestingly, the therapeutic properties of vitamin E, especially tocotrienols, have been shown to benefit various aspects of human health, including bone health [2,3]. A study in normal male rats has shown that vitamin E supplementation for four months is able to improve bone structural and biomechanical properties [4]. Vitamin E can promote bone health by enhancing osteoblast activity (cells that are responsible for bone formation), as well as by suppressing osteoclast activity (cells that are involved in bone resorption) [5,6].

The tocotrienols-rich fraction (TRF) contains approximately 70% of tocotrienols and about 30% of tocopherols. Previous studies have demonstrated that tocotrienols are superior to tocopherols in improving bone strength [7]. For instance, Ahmad et al. (2005) reported that rats supplemented with 100 mg/kg of a palm-oil–tocotrienol mixture were protected against the negative effect of ferric nitrilotriacetate (free radicals) on the bone histomorphometric parameters, i.e., trabecular bone volume and trabecular thickness. However, the same dose of α-tocopherol was not able to demonstrate similar findings [8]. In another study, it has also been proven that tocotrienols are more effective than tocopherols at improving the mineral apposition rate and bone formation rate in male rat models of nicotine-induced bone loss [9].

Other studies have also employed TRF as a potential treatment for various bone diseases. A previous study has reported that oral administration of TRF (dose 60 mg/kg) improves bone fracture healing in rats with ovariectomy-induced osteoporosis through a reduction in the levels of free radicals [10]. A more recent study has also shown that TRF (30 mg/kg body weight) alone and in combination with virgin coconut oil (4.2 mL/kg body weight) is able to repair bone loss in animal models of osteoporosis [11]. In addition, supplementation with TRF has been found to benefit inflammation-mediated bone loss, such as rheumatoid arthritis and osteoarthritis, as TRF successfully reduces the plasma levels of pro-inflammatory cytokines and C-reactive proteins and prevents cartilage degradation in rats [12,13].

Despite the various benefits of tocotrienols, their use as a potential adjuvant therapy is still limited due to their poor oral bioavailability. Previous studies involving human volunteers have reported that the time taken to achieve a peak plasma concentration of orally administered tocotrienols is between 3 h and 4 h after the meal, and the elimination half-life is between 2.3 h and 4.4 h [14,15]. In addition, studies have also shown that the tissue uptake of tocotrienols is attenuated in the presence of tocopherol [16]. This is due to the alpha-tocopherol transport protein (α-TTP), the main vitamin E transport protein in the circulation, having a higher affinity with α-tocopherol compared to the other vitamin E isoforms [16,17].

To address this issue, the application of nanotechnology is currently being explored for efficient delivery and improved bioavailability of tocotrienols [18]. Previous studies have demonstrated that the use of solid lipid nanoparticles (SLN) and nanostructured lipid carriers (NLC) to deliver tocotrienols not only enhances its anti-cancer properties, but also improves its oral bioavailability [19,20]. This is due to the enhanced intestinal permeability of tocotrienol following encapsulation into SLN [20]. Similarly, studies have also reported that tocotrienols prepared in the form of a self-emulsifying drug delivery system have improved oral bioavailability owing to the increased intestinal absorption of tocotrienols [21,22]. In addition, encapsulation of tocotrienols into polymeric nanoparticles using polymers such as polyethylene glycols, chitosan, or poly (lactic-co-glycolic) acid has also been shown to improve its cellular uptake, anti-proliferative activity, and oral bioavailability [23,24]. Taken together, the use of nanocarriers is crucial for a more efficient delivery of tocotrienols; therefore, the potential use of different forms of nanocarriers could be further explored.

Hydroxyapatite (Ca_10_(PO_4_)_6_(OH)_2_) is one of the most commonly used biomaterials in tissue engineering because it has a chemical and structural similarity to bone minerals. Recently, studies have expanded into the use of the nano-hydroxyapatite (nHA) as a potential carrier for various types of drugs or active compounds for targeted delivery to the bone [25]. nHA, typically, has a small size within the nanometer size range which allows rapid internalization, as well as providing a large surface area for the adsorption of drugs and/or active compounds and cells [26]. Most importantly, the nHA is non-toxic, as its degradation products, calcium (Ca^2+^) and phosphate ions (PO_4_^3−^), are inherent to the body and do not trigger an immune response [27]. A previous study has reported that the release of vitamin K (VK) from the hydroxyapatite/VK nanocomposite is significantly high and that hydroxyapatite possesses good biodegradation abilities for tissue engineering purposes [28]. A more recent study has developed a sublingual osteoporosis therapy (SLOT) by loading salmon calcitonin, an anti-resorptive agent, into hydroxyapatite nanoparticles for improved efficacy in a rat model of osteoporosis [29].

Nevertheless, the most important aspects of nanoparticle development are the design and optimization processes. To date, the most common approach or technique employed in nanoformulation design and optimization is the utilization of the central composite design (CCD) of the response surface methodology (RSM). CCD has been successfully used in previous studies involving nanoformulation optimizations and has proved to be a good and reliable model [30,31]. Briefly, CCD simultaneously evaluates the influence of several individual variables on the outcomes of an experiment [32]. The optimization procedure is performed using a Design of Experiments software, for example, Design-Expert^®^, which requires choosing the variables involved, formulating mathematical equations, and running a set of experiments in a randomized order by setting limits to the chosen variables [33]. The response surface contour plots are then generated to visualize the correlation between the selected variables, thus allowing the optimum experimental conditions to be chosen for the best possible outcomes.

To the best of our knowledge, no study has been published to date that establishes the nHA as a carrier to improve the oral bioavailability of the TRF. Therefore, this study aims to develop an nHA-encapsulating TRF (nHA/TRF) with an optimized condition for efficient drug delivery. The optimization process is performed by employing RSM, specifically CCD. The optimized nHA/TRF formulation is validated prior to further tests and its physical properties and morphology are also characterized.

## 2. Materials and Methods

### 2.1. Materials

The TRF (EVNol™ 50%) used in this study was gifted by ExcelVite Sdn. Bhd., Perak, Malaysia. The TRF was composed of about 11.3% α-tocopherol, 14.1% α-tocotrienol, 2.1% β-tocotrienol, 18.6% γ-tocotrienol, and 5.7% δ-tocotrienol. The nHA (nanopowder, <200 nm particle size, ≥97% synthetic) and D-mannitol were purchased from Sigma-Aldrich (St. Louis, MO, USA). The Milli-Q filtering system (Milli-Q Direct 16, Merck Millipore (Burlington, MA, USA) was used to deionize the water. All other solvents used were analytical-grade reagents.

### 2.2. Central Composite Design

A three-factor three-level central composite design was employed to investigate the effect of the independent variables, i.e., mass of the nHA (X_1_), concentration of the TRF (X_2_), and incubation time (X_3_), on three dependent variables, i.e., particle size (Y_1_), zeta potential (Y_2_), and encapsulation efficiency (Y_3_). A total of 20 CCD experiments were conducted using the Design-Expert^®^ software (version 6.0, Stat ease Inc., Minneapolis, MN, USA). The experimental design included six replicates of the center points, seven axial and seven factorial points, in a randomized order. The center points were repeated to ensure the reproducibility of the employed method. The data were analyzed using a response surface regression analysis. A polynomial model was chosen based on the significant terms (*p* < 0.05), the least significant lack of fit, the coefficient of variance, and the multiple correlation coefficient (R^2^), as provided by the Design-Expert^®^ software. The upper and lower limits of the independent variables are outlined in Table 1.

The optimal values of the independent variables for the nHA/TRF formulation were chosen based on the ideal responses, which are small particle size, high zeta potential values, and high encapsulation efficiency. The response surface behavior was analyzed for the response function (Y) using the polynomial Equation (1):

*Y_i_* = *β*_0_ + *β*_1_*X*_1_ + *β*_2_*X*_2_ + *β*_3_*X*_3_ + *β*_11_*X*_1_^2^ + *β*_22_*X*_2_^2^ + *β*_33_*X*_3_^3^ + *β*_12_*X*_1_*X*_2_ + *β*_13_*X*_1_*X*_3_ + *β*_23_*X*_2_*X*_3_ (1)

where *Y* is the predicted response, *X*_1_, *X*_2_, and *X*_3_ are the independent variables, and *β*_0_, *β*_1_, *β*_2_, and *β*_3_ are the coefficients associated with each variable. The analysis of variance (ANOVA) was used to determine the significance of the differences among the independent variables. All significant variables (*p* < 0.05) were included in the final reduced model. Three-dimensional (3D) response surface plots were generated to visualize the interaction effect between the variables and the responses. The R^2^ values should be greater than 0.8 to ensure the model has a good fit.

To validate and establish the final reduced model, the actual experimental values were compared with the predicted values calculated using the polynomial Equation (1).

### 2.3. Preparation of the nHA/TRF for RSM Optimization

**Figure 1:**
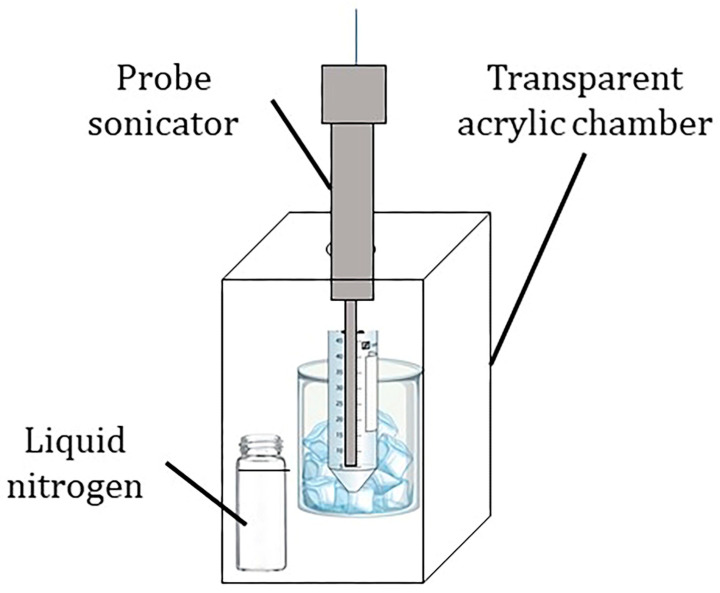
The experimental set-up for the ultrasonication process during the preparation of nHA/TRF.

The nHA/TRF was prepared by using the incubation method, where the nHA was suspended in a TRF solution diluted in n-hexane. The suspension was ultrasonicated using a probe sonicator (QSonica Q55, QSonica, Newtown, CT, USA) for 2 min at an 80% amplitude on ice. The ultrasonication process was performed in a customized chamber in the presence of liquid nitrogen to minimize the possibility of TRF oxidation (Figure 1). The dispersion was incubated at 20 °C in an incubator shaker (IKA KS 4000 i control, IKA, Staufen, Germany). The nHA/TRF dispersion was then subjected to high-speed centrifugation of 6000 rpm at 4 °C for 10 min (Himac CR22N, Hitachi Koki, Tokyo, Japan) to separate the pellet from the unentrapped TRF. The pellet was redispersed two times with n-hexane and the supernatants were collectively analyzed to estimate the TRF entrapment efficiency using a UV–Vis spectrophotometer (Shimadzu UV-2450, Shimadzu, Kyoto, Japan). The nHA/TRF pellet was dried using filter paper and crushed using a mortar and pestle to obtain a fine powder for size and zeta potential measurements. All procedures were carried out in a dark room, with minimal light exposure.

### 2.4. Preparation of the Freeze-Dried nHA/TRF

The nHA/TRF pellet was prepared using the same incubation method, with the optimized mass of nHA, the concentration of the TRF, and the incubation time. The isolated nHA/TRF pellet was freeze-dried after redispersing the pellet in 5 mL of filtered distilled water, with mannitol (10% *w*/*v*) used as a cryoprotectant. The concentration of mannitol was previously determined through a separate experiment (unpublished data). The dispersion was frozen in liquid nitrogen and stored at −80 °C for 24 h prior to freeze drying. Sublimation lasted for 48 h at a vacuum pressure of 10–50 mbar, with the condenser temperature maintained at less than −55 °C (FreeZone 4.5L, LABCONCO, Kansas City, MO, USA). The freeze-dried nHA/TRF was stored in an amber screw cap glass bottle containing a food-grade desiccant for further characterization.

### 2.5. Particle Size, Zeta Potential, and Polydispersity Index

Particle size, zeta potential, and polydispersity index (PDI) measurements were performed using a particle size analyzer (Malvern Nano ZS90, Malvern Panalytical, Malvern, UK). The nHA/TRF was redispersed in deionized water (0.5 mg/mL) with 1:5 and 1:2 dilution ratios for the non-freeze-dried and freeze-dried nHA/TRF, respectively. The diluted samples were placed in disposable cuvettes for size and PDI measurements or injected into a folded capillary cell for zeta potential measurements. All measurements of the nHA/TRF samples were performed in triplicates.

### 2.6. Entrapment Efficiency

The entrapment efficiency was determined by separating the unentrapped TRF from the nHA/TRF via high-speed centrifugation. The supernatant containing the unentrapped TRF was quantified using a UV–Vis spectrophotometer at a 295 nm wavelength, as demonstrated in previous research [19]. The percentage of the entrapment efficiency (%EE) was calculated using the following formula;

$$\%EE=\frac{Initial\;amount\;of\;drug\;\left(\mathrm{mg}\right)-Amount\;of\;unentrapped\;drug\;(\mathrm{mg})}{Initial\;amount\;of\;drug\;(\mathrm{mg})}\;\times \;100$$

where the initial amount of the drug is the amount of the drug initially incubated with the nHA, and the unentrapped drug is the amount of the drug present in the supernatant following the high-speed centrifugation and washing processes.

### 2.7. Transmission Electron Microscopy

The samples were visualized using a high-resolution transmission electron microscope (JEM 2100F, JEOL Ltd., Tokyo, Japan). The samples were diluted 1:100 in deionized water prior to transmission electron microscopy (TEM) observation. A drop of sample was deposited on a 3 mm carbon-coated copper grid. The samples were negatively stained with a 2% uranyl acetate solution (% *w*/*v*) for 2 min, and the excess liquid was blotted up using filter paper. The samples were then left to dry at room temperature before observation.

### 2.8. Statistical Analyses

Statistical analyses were performed using the GraphPad PRISM software (version 10, USA). The data obtained were expressed as the mean ± the standard error of the mean (SEM). A student’s *t*-test was used to evaluate the statistical significance, where a *p*-value lower than 0.05 (*p* < 0.05) was considered to be statistically significant.

## 3. Results and Discussion

### 3.1. Fitting the Response Surface Model

A total of 20 experiments were automatically generated in a randomized order. The mean particle size, zeta potential, and encapsulation efficiency are outlined in Table 2. All the experiments showed a particle size within the nanometer range of 220.4–316 nm. Meanwhile, all the experiments showed negative zeta potential values ranging between −13.2 mV and −19.8 mV. The encapsulation efficiency values were in the range of 4–33.4%. Six center points (highlighted in gray in Table 2) with a fixed nHA mass, concentration of the TRF, and incubation time were performed to ensure the reproducibility and consistency of the results.

All data were statistically analyzed and the best-fit model for the independent variables was identified through the regression equations, coefficients of multiple determinations (R^2^), adequate precision values, F-value, probability values (*p*-value), and *p*-value for the lack-of-fit (Table 3). Previous studies have reported that a high F-value and a low *p*-value indicate a significant interaction between the tested variables [30,34]. All factors showed a significant effect on particle size and zeta potential, with small *p*-values < 0.05, and encapsulation efficiency, with a *p*-value < 0.0001. These factors also showed good R^2^ values (R^2^ > 0.8), which indicate that the quadratic polynomial models are significant and sufficient to represent the actual interaction between independent and dependent variables [31]. The R^2^ values for the particle size, zeta potential, and encapsulation efficiency were 0.8632, 0.8181, and 0.9607, respectively. These values indicate that the model can predict up to 86.32% of the particle size, 81.81% of the zeta potential, and 96.07% of the encapsulation efficiency observed values, leading to a high correlation between the independent and dependent variables. In addition, adequate precision measures the signal-to-noise ratio, whereby a ratio of more than 4 is desired for a well-fitting model [34,35]. The adequate precision values reported herein are 9.136, 6.141, and 17.418 for the particle size, zeta potential, and encapsulation efficiency, respectively. Essentially, the *p*-values for the lack-of-fit in relation to all dependent variables were high (>0.05), indicating that the model has a good fit. Therefore, the quadratic model obtained was fitted to the data to obtain the predicted responses.

The positive or negative coefficient values associated with each independent variable (A, B, and C) represent the tendency and magnitude of the terms influencing the dependent variables [34,35]. The equations showed that the mass of the nHA and the concentration of the TRF had a positive effect on particle size, while incubation time had a negative effect (Table 3). Conversely, the mass of the nHA and the concentration of the TRF had a negative effect on the zeta potential, while incubation time had a positive effect. On the other hand, the mass of the nHA and the incubation time positively affected the encapsulation efficiency, while the concentration of the TRF had a negative effect.

**Figure 2:**
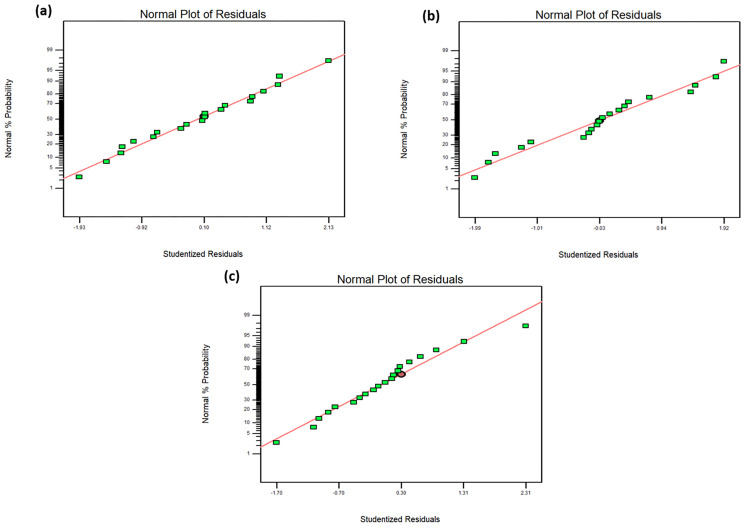
The normal probability plot of the residuals of (**a**) particle size, (**b**) zeta potential, and (**c**) encapsulation efficiency, indicating the normally distributed experimental values.

The normal probability plot of the residuals of particle size, zeta potential, and encapsulation efficiency are plotted in Figure 2. The experimental values obtained mostly fall on a straight line, indicating that these values are normally distributed.

### 3.2. Influence of the Independent Variables on the Particle Size of the nHA/TRF

**Figure 3:**
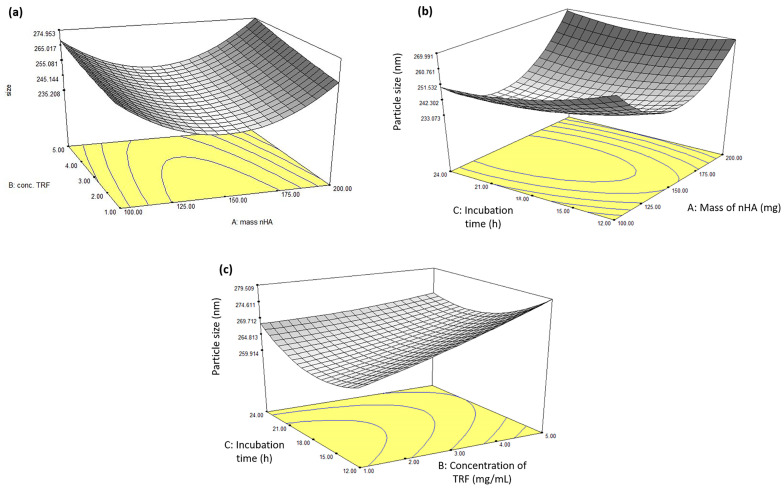
Response surface plots showing the effect of the interaction between the (**a**) mass of the nHA and the concentration of the TRF with a fixed incubation time of 14.23 h, (**b**) mass of the nHA and the incubation time with a fixed TRF concentration of 2.96 mg/mL, and (**c**) concentration of the TRF and the incubation time with a fixed nHA mass of 200 mg on the particle size of the nHA/TRF.

The three-dimensional (3D) response surface plots for the interaction between the independent variables and the particle size of the nHA/TRF are depicted in Figure 3. At the highest and lowest concentrations of TRF, a decrease in particle size can be seen at increasing masses of nHA, up to 150 mg (Figure 3a). Similarly, at the highest and lowest incubation time, the smallest particle sizes were obtained with increasing masses of nHA, up to 150 mg (Figure 3b). On the other hand, at a fixed nHA mass of 200 mg, an increase in the concentration of the TRF after 12 h of incubation clearly increased the particle size (Figure 3c). The increased particle size of the nHA/TRF, in conjunction with the increased concentration of the TRF, was probably due to TRF adsorption on the surface of nHA. A previous study has reported that the incorporation of the vitamin K causes an increased particle size due to drug adsorption onto the surface of the nHA nanocomposite [36]. With regard to this, an increased incubation time resulted in increased particle sizes, probably due to the extra time allowing for the adsorption of TRF on the surface of nHA.

### 3.3. Influence of the Independent Variables on the Zeta Potential of the nHA/TRF

**Figure 4:**
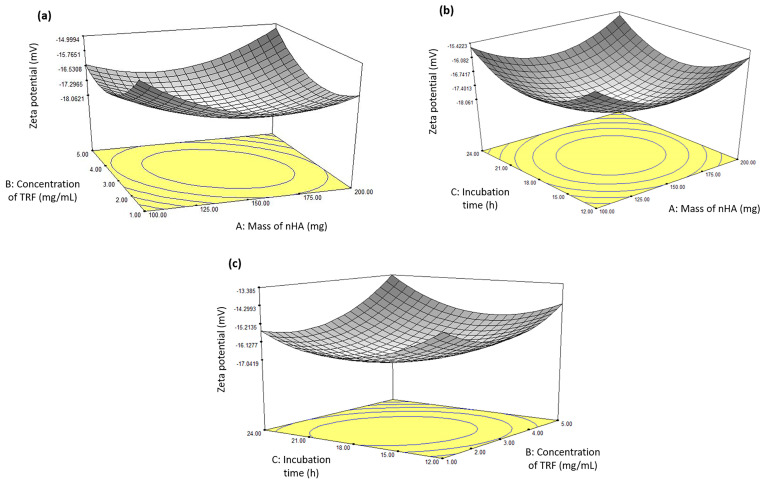
Response surface plots showing the effect of the interaction between the (**a**) mass of the nHA and the concentration of the TRF with a fixed incubation time of 14.23 h, (**b**) mass of the nHA and the incubation time with a fixed TRF concentration of 2.96 mg/mL, and (**c**) concentration of the TRF and the incubation time with a fixed nHA mass of 200 mg on the zeta potential of the nHA/TRF.

The 3D response surface plots for the interaction between the independent variables and the zeta potential of the nHA/TRF were also plotted (Figure 4). The zeta potential is a measure of the electrostatic repulsion occurring among dispersed nanoparticles and is an indication of dispersion stability [37]. It is recommended for nanoparticles to have a zeta potential value of more than ±15 mV for the dispersion to be stable [38]. Under a fixed nHA mass of 150 mg and a fixed incubation time of 14.23 h, a more negative zeta potential was observed with increasing concentrations of TRF, up to 3 mg/mL, beyond which it started to decrease (Figure 4a). Under a fixed nHA mass of 150 mg, increasing the incubation time to up to 18 h resulted in an increased zeta potential when the TRF concentration was fixed at 2.96 mg/mL (Figure 4b). Under a fixed nHA mass of 200 mg, the highest zeta potential value was obtained at the middle values of the TRF concentration and incubation time (Figure 4c).

### 3.4. Influence of the Independent Variables on the Encapsulation Efficiency of the nHA/TRF

**Figure 5:**
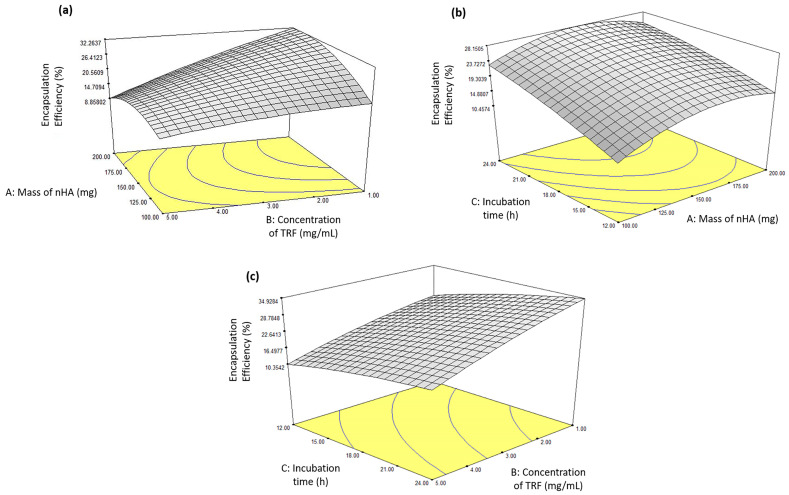
Response surface plots showing the effect of the interaction between the (**a**) mass of the nHA and the concentration of the TRF with a fixed incubation time of 14.23 h, (**b**) mass of the nHA and the incubation time with a fixed TRF concentration of 2.96 mg/mL, and (**c**) concentration of the TRF and the incubation time with a fixed nHa mass of 200 mg on the encapsulation efficiency of the nHA/TRF.

The 3D surface plots for the interaction between the independent variables and the encapsulation efficiency of the nHA/TRF are shown in Figure 5. Under an nHA mass of 150 mg and an incubation time of 14.23 h, increasing the concentration of the TRF reduced the encapsulation efficiency (Figure 5a). Meanwhile, an increased incubation time under an nHA mass of 150 mg and a fixed TRF concentration of 2.96 mg/mL resulted in an increased encapsulation efficiency (Figure 5b). Interestingly, increasing the incubation time for the highest concentration of the TRF with a fixed nHa mass of 200 mg did not significantly increase the encapsulation efficiency (Figure 5c). On the other hand, decreasing the concentration of the TRF under a fixed nHA mass of 200 mg after 18 h of incubation significantly increased the encapsulation efficiency (Figure 5c). The reduction in the encapsulation efficiency at higher concentrations of the drug was probably due to the repulsion between the adsorbed drug on the nHA particles and the drug molecules in the solution [33].

### 3.5. Verification of the Final Reduced Model

Based on the final CCD outcomes, the optimum mass of nHA, the optimum concentration of the TRF, and the optimum incubation time were automatically calculated using the polynomial equations presented in Table 3. Five different solutions were suggested, and the first suggestion was selected for validation due to its highest desirability value (Table 4).

The proposed optimum nHA/TRF composition and incubation time were validated by comparing the predicted and experimental values of each dependent variable (Table 5). No significant differences were observed between the experimental and the predicted values with respect to the particle size, the zeta potential, and the encapsulation efficiency, indicating that the data obtained are in good agreement with the predicted outcomes.

### 3.6. Physical Characteristics and Encapsulation Efficiency

**Figure 6:**
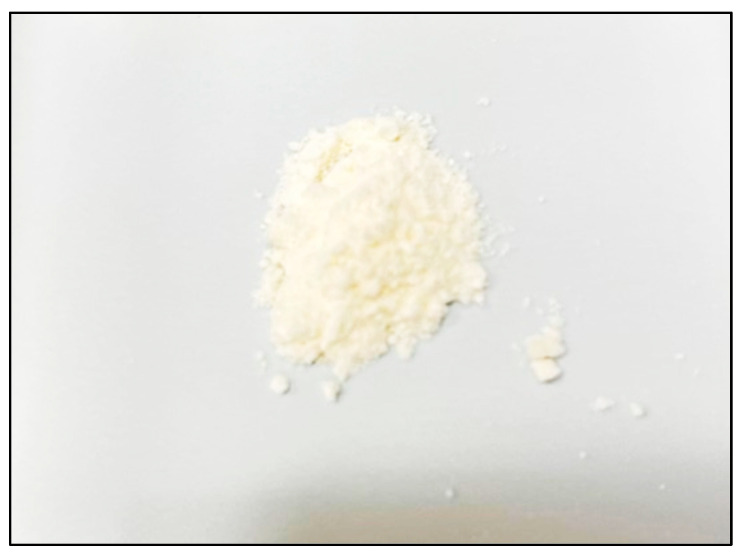
Physical appearance of the freeze-dried nHA/TRF with 10% mannitol (% *w*/*v*).

The freeze-drying process was performed to remove all solvents, prevent the aggregation of the samples, and ensure the stability of the nanoparticles [39]. In this study, the freeze-drying process in the presence of 10% mannitol (% *w*/*v*) resulted in a fine nHA/TRF nanopowder (Figure 6). A previous study has also shown that solid lipid nanoparticles lyophilized in the presence of 10% mannitol resulted in a fine powder that was fully re-dispersed within 30 s of vortexing [40].

**Figure 7:**
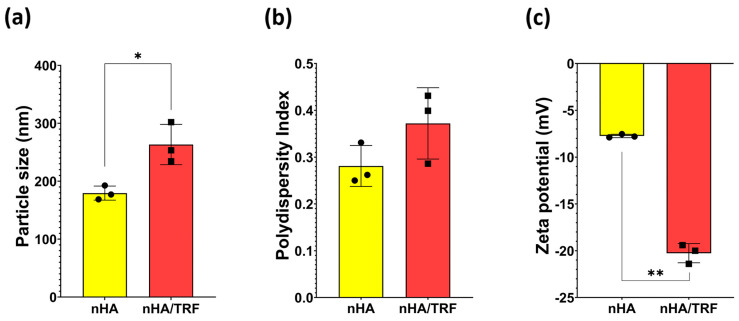
Particle size (**a**), polydispersity index (**b**), and zeta potential (**c**) of the unloaded nHA and nHA/TRF. Data expressed as the mean ± SEM (*n* = 3) (* *p* < 0.05; ** *p* < 0.001).

The mean particle size and zeta potential of the nHA/TRF measured by dynamic light scattering (DLS) were significantly higher compared to those of the blank nHA (Figure 7a,c). Previous studies employing drug-loaded nHA-based nanoparticles have also reported an increase in particle size and zeta potential values compared to the nHA nanoparticles alone [27,41]. The increase in the particle size of the nHA/TRF was due to the adsorption of the TRF on the surface of nHA. Meanwhile, the increase in the zeta potential was probably due to changes in the surface charge of the nHA following TRF adsorption. Previous studies have reported that the integration of the vitamin D3 with a hydroxyapatite-based scaffold resulted in a more negative zeta potential, possibly due to the formation of hydrogen bonds between the vitamin D3 and the other components of the scaffold [42]. However, no significant difference was observed with respect to the polydispersity index between the nHA/TRF and the nHA (Figure 7b). This indicates that the nHA/TRF particle has an improved stability and is uniformly distributed.

The encapsulation efficiency percentage of the nHA/TRF was approximately 18.1%. This low encapsulation efficiency has also been observed in relation to other nHA particles loaded with lipid-soluble vitamins in the absence of other polymers. A recent study employing vitamin-D-loaded hydroxyapatite nanoparticles has reported a low loading capacity of 10.86% [43]. Moreover, although the porosity of nHA particles may affect the encapsulation of the TRF, it was not measured in this study. A previous study has shown that the morphology, surface area, and porosity of the nHA may affect the loading efficiency of drugs or active compounds [44].

### 3.7. Morphological Observations Using Transmission Electron Microscopy

**Figure 8:**
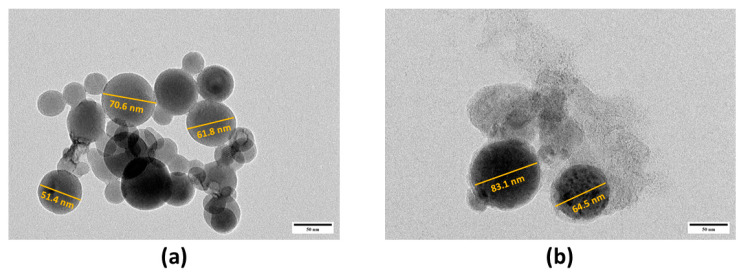
Transmission electron micrograph of the (**a**) unloaded nHA and (**b**) nHA/TRF at 50,000× magnification.

TEM in Figure 8a revealed that the nHA particles have a spherical structure with high agglomeration, consistent with a previous study [45]. On the other hand, the nHA/TRF maintained its spherical structure and appeared to be less agglomerated (Figure 8b). This observation corresponded to the increased value of the zeta potential possessed by the nHA/TRF and obtained from DLS, as a higher negative surface charge value enhances the electrostatic repulsion between particles, preventing them from aggregating and promoting a more stable distribution [30]. All samples showed a particle size within the nanometer range (<300 nm), consistent with the data obtained from DLS.

## 4. Conclusions

The formulation optimization of the nHA/TRF particle using the RSM method successfully produced a nanosized carrier for the oral administration of the TRF. Moreover, the interaction between the independent and dependent variables was well-understood from the response surface plots generated in this study. The formulation was validated by comparing the experimental and predicted outcomes, whereby no significant difference was observed. Therefore, the optimized composition and incubation time of the nHA/TRF suggested by the RSM were satisfactory. The physical properties of the nHA/TRF revealed that the particle size is within the nanometer size range, with a narrow PDI and an acceptable zeta potential and encapsulation efficiency. The fine nHA/TRF powder produced was easily re-dispersible in water and has a spherical morphology. Future studies are warranted to further evaluate the physicochemical properties of the nHA/TRF particles, especially their release kinetics, chemical composition, and thermal properties. The potential application of the nHA/TRF in nutraceuticals can also be investigated by observing the efficacy and oral bioavailability of the nHA/TRF in vivo. In conclusion, the nHA was established as a nanosized delivery vehicle for the TRF as shown by its promising physical properties.

## Figures and Tables

**Table 1:** The coded levels of the low, medium, and high limits of the independent variables used in the RSM.

Independent Variables	Coded Levels		
	Low	Medium	High
X_1_: mass of the nHA (mg)	100	150	200
X_2_: concentration of the TRF (mg/mL)	1	3	5
X_3_: incubation time (h)	12	18	24

Abbreviations: nHA, nano-hydroxyapatite; TRF, tocotrienols-rich fraction.

**Table 2:** Central composite design (CCD) for all the experimental runs with the independent and dependent variables.

		Independent Variables			Dependent Variables		
Run	Type	Mass of the nHA (mg)	Concentration of the TRF (mg/mL)	Incubation Time (h)	Particle Size (nm)	Zeta Potential (mV)	Encapsulation Efficiency (%)
1	Fact	200	5	12	271.9	−15.0	4.4
2	Axial	65.91	3	18	295.2	−14.0	5.1
3	Fact	100	5	24	257.9	−15.1	17.6
4	Centre	150	3	18	235.7	−19.8	25.6
5	Fact	100	1	12	253.9	−13.9	13.2
6	Centre	150	3	18	250.0	−15.7	23.4
7	Centre	150	3	18	240.0	−17.8	23.7
8	Axial	150	3	28.09	225.4	−14.6	31.9
9	Fact	100	1	24	258.7	−14.2	25.9
10	Centre	150	3	18	236.2	−17.9	21.3
11	Fact	100	5	12	274.0	−16.0	10.1
12	Centre	150	3	18	226.5	−19.1	24.5
13	Axial	150	3	7.91	274.8	−13.5	9.9
14	Centre	150	3	18	220.4	−18.2	24.7
15	Fact	200	1	24	269.0	−15.6	33.4
16	Axial	150	−0.36	18	238.1	−14.6	32.6
17	Fact	200	1	12	253.7	−15.1	26.2
18	Fact	200	5	24	281.3	−13.2	9.1
19	Axial	234.09	3	18	316.0	−14.7	18.0
20	Axial	150	6.36	18	238.4	−14.6	8.1

Abbreviations: nHA, nano-hydroxyapatite; TRF, tocotrienols-rich fraction.

**Table 3:** Analysis of variance (ANOVA) of the regression coefficients of the quadratic equations for particle size, zeta potential, and encapsulation efficiency. The asterisk (*) indicates significant terms (*p* < 0.05).

Variables	Particle Size, nm (Y_1_)		Zeta Potential, mV (Y_2_)		Encapsulation Efficiency, % (Y_3_)	
	F-Value	*p*-Value	F-Value	*p*-Value	F-Value	*p*-Value
Model	7.01	0.0027 *	5.00	0.0096 *	27.15	<0.0001 *
Linear terms						
A	2.02	0.1860	0.045	0.8367	8.96	0.0135 *
B	1.16	0.3072	0.015	0.9064	111.36	<0.0001 *
C	2.22	0.1669	0.00015	0.9906	54.57	<0.0001 *
Quadratic terms						
A^2^	55.09	<0.0001 *	15.98	0.0025 *	41.56	<0.0001 *
B^2^	0.077	0.7876	13.68	0.0041 *	3.17	0.1054
C^2^	2.35	0.1561	18.98	0.0014 *	2.22	0.1675
Interaction terms						
AB	0.098	0.7607	3.00	0.1137	23.49	0.0007 *
AC	1.01	0.3381	0.049	0.8298	1.34	0.2732
BC	0.56	0.4711	1.22	0.2959	1.16	0.3074
Other parameters						
Lack-of-fit	1.98	0.2358	0.29	0.9023	4.86	0.0538
R^2^	0.8632		0.8181		0.9607	
Adequate precision	9.136		6.141		17.418	
Second order polynomial equation	Y_1_ = 234.85 + 4.86A + 3.68B − 5.10C + 24.73A2 + 0.92B^2^ + 5.11C^2^ + 1.40AB + 4.50AC − 3.35BC		Y_2_ = −18.06 − 0.064A − 0.037B + 0.0037C + 1.18A^2^ + 1.09B^2^ + 1.29C^2^ + 0.69AB + 0.087AC + 0.44BC		Y_3_ = 23.86 + 2.05A − 7.23B + 5.06C − 4.30A^2^ − 1.19B^2^ − 0.99C^2^ − 4.34AB − 1.04AC − 0.96BC	

Independent variable A: mass of nHA; B: concentration of the TRF; C: incubation time.

**Table 4:** Solutions generated by the CCD according to the final reduced model.

No.	Mass of the nHA (mg)	Concentration of the TRF (mg/mL)	Incubation Time (h)	Desirability
1	200.00	2.96	14.23	0.991
2	200.00	3.61	19.57	0.986
3	199.98	3.06	14.30	0.982
4	100.00	2.39	18.59	0.884
5	100.13	2.30	18.46	0.879

Abbreviations: nHA, nano-hydroxyapatite; TRF, tocotrienols-rich fraction.

**Table 5:** The predicted and observed experimental values for the optimized nHA/TRF nanoparticle. Data for the experimental values are expressed as the mean ± SEM (*n* = 3).

Dependent Variables	Predicted Values	Experimental Values
Particle size (nm)	266.7	268.1 ± 9.29
Zeta potential (mV)	−16.5	−16 ± 0.60
Encapsulation efficiency (%)	18.9	18.1 ± 0.73

## Data Availability

The original contributions presented in this study are included in the article. Further inquiries can be directed to the corresponding authors.
